# Icariin exerts anti-tumor activity by inducing autophagy via AMPK/mTOR/ULK1 pathway in triple-negative breast cancer

**DOI:** 10.1186/s12935-024-03266-9

**Published:** 2024-02-14

**Authors:** Mei Zhao, Panling Xu, Wenjing Shi, Juan Wang, Ting Wang, Ping Li

**Affiliations:** 1 Department of Chinese Integrative Medicine Oncology, The First Affiliated Hospital of Anhui Medical University, 120 Wanshui Road, Hefei, 230032 Anhui People’s Republic of China; 2 Department of Integrated Traditional Chinese and Western Medicine, Anhui Medical University, Hefei, China; 3 Graduate School of Anhui University of Traditional Chinese Medicine, Hefei, China

**Keywords:** Icariin, TNBC, Autophagy, Apoptosis, AMPK

## Abstract

**Background:**

Breast cancer is the most prevalent female tumor, of which triple-negative breast cancer (TNBC) accounts for about 15%. Characterized by its aggressive nature and limited treatment options, TNBC currently stands as a significant clinical challenge. This study aimed to investigate the effects of icariin (ICA) on TNBC and explore the underlying molecular mechanism.

**Methods:**

Cell viability was assessed using CCK-8 assay, whereas the impact of ICA on cell proliferation was determined using colony formation assay and detection of proliferating cell nuclear antigen protein. Wound healing and transwell assays were used to evaluate the effects of ICA on cell migration and invasion, respectively. Flow cytometry was used to analyze cell cycle distribution and apoptosis. Transmission electron microscopy and monodansylcaverine staining were performed to detect the induction of autophagy, whereas molecular docking was conducted to predict the potential targets associated with autophagy. The in vivo anti-tumor effects of ICA were evaluated using a TNBC 4T1 xenograft mouse model. Protein expression levels were examined using immunoblotting and immunohistochemistry.

**Results:**

In vitro, ICA effectively suppressed the viability, proliferation, migration, and invasion of TNBC cells and induced G0/G1 phase cell cycle arrest, apoptosis, and autophagy in TNBC cells by regulating the adenosine monophosphate-activated protein kinase (AMPK)/mammalian target of rapamycin (mTOR)/Unc-51-like kinase 1 (ULK1) signaling pathway. The knockdown of AMPK and inhibition of autophagy with 3-methyladenine reversed the effects of ICA, highlighting the importance of AMPK and autophagy in the anti-cancer mechanism of ICA. In vivo, ICA significantly inhibited TNBC growth, promoted autophagy, and regulated AMPK/mTOR/ULK1 pathway.

**Conclusions:**

Our findings demonstrated that ICA exerts anti-cancer effects against TNBC and the associated molecular mechanisms. This study will help to facilitate further preclinical and clinical investigations for the treatment of TNBC.

## Background

Breast cancer (BC) is associated with the highest incidence of malignancy in females, with up to 2.3 million new cases reported in 2020 [1]. Triple-negative breast cancer (TNBC), which accounts for approximately 15% of all BC cases, is characterized by the absence of estrogen receptors, progesterone receptors, and human epidermal growth factor receptor 2 expression. Tumors of this subtype are highly heterogeneous and aggressive, often associated with early recurrence, and preferentially occur in young females, especially those of Asian and African descent [2–4]. The unique biological characteristics of TNBC hinder its treatment. Although chemotherapy remains the primary treatment for TNBC, significant side effects and drug resistance limit its efficacy [5]. Therefore, alternative treatment for TNBC remains warranted.

Autophagy is a cellular process that maintains cellular homeostasis by recycling damaged organelles and misfolded proteins [6, 7]. In cancer, autophagy exhibits complex and contextually relevant functions associated with tumor suppression, promotion, or therapy resistance. In early clinical trials, potent autophagy inducers, such as tamoxifen and everolimus, as well as autophagy inhibitors, such as chloroquine, approved by the U.S. Food and Drug Administration, have been used in combination with chemotherapy or radiation to increase tumor cell death or restore chemotherapy sensitivity [8, 9]. Several recent studies have linked autophagy to TNBC, making it a potential target for TNBC therapy.

Some Chinese herbal medicines and their extracts have been found to have the potential to regulate autophagic pathways and, therefore, have anti-cancer treatment potential. For example, artemisinin induces autophagy in cancer cells, inducing cell death [10]. Icariin (ICA) is a flavonoid extracted from Epimedium with potent anti-proliferative effects against hepatocellular carcinoma, pancreatic cancer, and BC [11–14]. However, the molecular mechanism underlying the effects of ICA on autophagy-mediated anti-TNBC activity remains unclear.

Therefore, this study investigates the anti-cancer effects of ICA on TNBC and its underlying mechanisms. We found that ICA inhibited cell viability, proliferation, and invasion, blocked the cell cycle, and inhibited autophagy and apoptosis in TNBC cells. Furthermore, ICA promoted cellular autophagy by activating the adenosine monophosphate-activated protein kinase (AMPK)/mammalian target of rapamycin (mTOR)/Unc-51-like kinase 1 (ULK1) signaling pathway. Our findings suggest ICA as a natural product acting on autophagy with anti-tumor activity against TNBC.

## Methods

### Cells and reagents

Human TNBC cell lines MDA-MB-468 (468) and MDA-MB-231 (231), and mouse TNBC cell lines 4T1 were purchased from the National Collection of Authenticated Cell Cultures (Shanghai, China) and identified using short tandem repeat sequence analysis. Cells were cultured in DMEM medium (BI, USA) containing 10% fetal bovine serum (BI, USA) and 1% penicillin–streptomycin solution (100 × , Beyotime, China) in a humid incubator with 5% CO_2_ at 37 °C. ICA (purity > 98%, as measured using high-performance liquid chromatography) was purchased from Shanghai Yuanye Bio-Technology Co., Ltd. (China). ICA was dissolved in 100 mM of 100% dimethyl sulfoxide (DMSO) and stored at − 20 °C. The medium used contained no more than 0.1% DMSO. Metformin (MET) and 3-Methyladenine (3-MA) were purchased from MedChemExpress (USA) and configured with phosphate buffer saline (PBS) when used.

### Cell viability assay

Suspensions of 468, 231, and 4T1 cells were seeded in 96-well plates at a concentration of 1 × 10^4^ cells/well and incubated for 24 h. After adherence, cells were treated with different concentrations of ICA (0, 12.5, 25, 50, 75, and 100 µmol/L) and incubated at 37 °C for 24, 48, or 72 h. Cell viability was measured using an enhanced Cell Counting Kit-8 (CCK-8) (Beyotime, China). Cells were stored in a solution containing CCK-8 DMEM (10 μL CCK-8 at 100 μL DMEM) for 2 h at 37 °C. Absorbance at 450 nm was measured using a microplate reader (Biotek Synergy H1, USA).

### Colony formation assay

The 468 and 4T1 cells were inoculated in 6-well plates (400–600 cells/well) for 48 h and then treated with different concentrations of ICA for 24 h. Following a 10-day incubation, colonies containing > 50 cells were fixed with 4% paraformaldehyde for 15 min, stained with 0.5% crystal violet for 20 min, imaged, and counted using ImageJ software (Germany). The experiment was repeated three times.

### Wound healing assay

Cells were inoculated into 6-well plates and scratched with the tip of a 1-mL pipette when the cells reached approximately 90% confluence. Cells were treated with different concentrations of ICA and cultured for 48 h. After scratching at 0, 24, and 48 h, cells were imaged using a microscope (Olympus, Japan). Wound closure was measured using ImageJ software.

### Transwell invasion assay

To assess the effect of ICA on cell invasion, cell culture chambers (Corning, NY, USA) were placed in a 24-well plate to separate the upper and lower chambers. Then, 1 × 10^5^ DMEM cell suspensions pretreated with different concentrations of ICA for 24 h were seeded into the upper chamber pre-coated with Matrigel, and a medium containing 10% FBS was placed in the lower chamber. Incubation at 37 °C for 24 h, non-invasive cells were wiped off the filter's upper surface using a cotton swab. On the bottom surface, invasive cells were fixed with 4% paraformaldehyde and stained with 0.1% crystal violet. Finally, the cells were imaged and counted under a microscope (Olympus, Japan).

### Cell cycle assay

Cell cycle analysis was performed using a Cell Cycle and Apoptosis Analysis Kit (Beyotime, China). Cells were collected following treatment with ICA and fixed in 70% ethanol at 4 °C for 24 h. A propidium iodide staining solution was applied to the cell samples, and they were incubated in the dark for 30 min at 37 °C. Flow cytometry (Beckman, USA) was used to detect the cell cycle, and Modfit software (version 5.0, USA) was used to analyze it.

### Cell apoptosis assay

The Annexin V-FITC Apoptosis Detection Kit (Beyotime, China) was used to detect cell apoptosis. Cells were stained with Annexin V-FITC/PI and analyzed using flow cytometry after being treated with ICA, ICA ± 3-MA, or ICA ± MET. Apoptotic cells were considered to be Annexin V + /PI + .

### Autophagy staining assay

An Autophagy Staining Assay Kit with monodansylcaverine (MDC) (Beyotime, China) was used to detect autophagy. Cells treated with different concentrations were incubated with MDC staining solution at 37 °C in the dark for 30 min. The green fluorescence observed using a fluorescence microscope (Olympus, Japan) indicated an autophagosome.

### Western blot analysis

Cells were treated with ICA for 24 h and then were lysed with RIPA lysis buffer containing phosphatase inhibitor and phenylmethylsulfonyl fluoride, and protein samples were separated using 12.5%, 10%, or 6% SDS-PAGE gels and transferred to PVDF membranes. Tumor tissues from mice were immersed in RIPA buffer, ground using a tissue homogenizer (JINGXIN CO.,LTD, China), and centrifuged to obtain the supernatant, which was referred to as tissue protein samples. After blocking with QuickBlock^™^ Blocking Buffer (Beyotime, China) for 20 min at room temperature, the membrane was incubated overnight with the primary antibody (1:1000) at 4 °C and incubated at room temperature for 1 h with the second antibody (HRP-labeled Goat Anti-Rabbit IgG(H + L), 1:5000). The target blots were then obtained using a highly sensitive enhanced chemiluminescence reagent (Beyotime, China) on the machine (Bio-Rad, USA). Antibodies against β-actin (AF5003), LC3B (AL221), AMPK alpha 1 (AF1627), and phospho-AMPK alpha 1 (Ser496) (AF2677) were purchased from Beyotime Company (China); phospho-ULK1 (Ser757) [Ser758] (AF4387), mTOR (AF6308), and phospho-mTOR (Ser2481) (AF3309) were purchased from Affinity company (China); proliferating cell nuclear antigen (PCNA; 13110) was purchased from Cell Signaling Technology (USA); BECN1 (T55092F), P62 (T55546F), and ULK1 (T5692F) were gifted by Abmart company (China); and the second antibody HRP-labeled Goat Anti-Rabbit IgG(H + L) (A0208) was purchased from Beyotime Company (China).

### Molecular docking

The structures of AMPK (PDB ID:4CFH), mTOR (PDB ID:3JBZ), and ULK1 (PDB ID:4WNO) were obtained from the PDB database (https://www.pdbus.org/). PyMol 2.5.0a0 software (Schrödinger, USA) was used to remove water molecules and irrelevant ligands from the target protein structures using AMPK, mTOR, and ULK1 proteins as receptors. The ICA structure was obtained from PubChem (https://pubchem.ncbi.nlm.nih.gov). The receptors were docked to the ligand ICA following hydrogenation, and atomic charges were added using AutoDock Tools-1.5.7 software (Scripps Research, USA). Molecular docking was carried out using AutoDock AutoDock4.2.6 software (Scripps Research, USA). PyMOL 2.5.0a0 was used to observe the binding of ICA to AMPK, mTOR, and ULK1.

### Knockdown of AMPK

The small interfering RNA of AMPK (si-AMPK) and negative control (si-NC) were designed and synthesized by GenePharma Co., Ltd (China). Approximately 1 × 10^5^ cells were seeded per well in a 6-well plate and cultured until reaching 50–60% confluence. Transfection was performed using Lipo8000^™^ Transfection Reagent (Beyotime, China) along with si-AMPK or si-NC (100 pmol/well). After 12 h, the medium was replaced, and cells were further cultured for an additional 36 h. Then, the cells underwent intervention with ICA (25 µM) for 24 h.

### In vivo tumor xenograft models

Five-week-old female BALB/c mice were purchased from Huachuang Sino Medicine Technology Co. Ltd. (SCXK2020-0009). All animal procedures were performed in accordance with the China Animal Welfare Guidelines and approved by the Animal Ethics Committee of Anhui Medical University (LLSC20160112). After 1 week of adaptation, 5 × 10^6^ 4T1 cells were subcutaneously injected into the right lower back of each mouse. The mice were randomly divided into three groups (control, 20, and 40 mg/kg) (n = 6) when the tumor volume reached approximately 100 mm^3^ (after approximately 1 week). Then, the daily injections of ICA were given intraperitoneally to mice in different groups. The tumor size and body weight of each mouse were measured every 3 days. The tumor volume was determined using digital Vernier calipers according to the formula: V (mm^3^) = 1/2 × length × width^2^. After 15 days, the mice were anesthetized and sacrificed, and tumor tissues were collected, measured, and imaged. Tumor tissues and main organs were fixed in 4% paraformaldehyde for subsequent experiments.

### Hematoxylin and eosin (H&E) staining and immunohistochemistry (IHC)

After fixation for at least 24 h, the tissues were dehydrated with ethanol solution and xylene, embedded in paraffin, and cut into 5-µm sections. Sections were rehydrated with ethanol and stained with hematoxylin (Sigma, USA) and eosin (Sigma, USA). Other sections were dewaxed and rehydrated, and antigens were extracted with 0.01 M sodium citrate buffer (pH 6.0). The sections were sequentially incubated with 3% hydrogen peroxide, 0.1% Triton X-100, and 3% BSA and treated with primary antibodies against Ki67, LC3B, or P62 for 2 h at 37 °C, followed by secondary antibodies. Finally, the sections were treated with 3,3ʹ-diaminobenzidine tetrahydrochloride and hematoxylin, dehydrated, dried, and then observed under a microscope.

### Statistical analysis

The results are presented as mean ± standard deviation (SD) from at least three independent experiments. One-way analysis of variance (ANOVA) (Dunnett’s post-hoc test) and Student's *t-*test were used for data analysis. P < 0.05 were considered statistically significant, recorded as *P < 0.05, **P < 0.01, and ***P < 0.001.

## Results

### ICA effectively inhibited viability and proliferation of TNBC cells in vitro

**Fig. 1:**
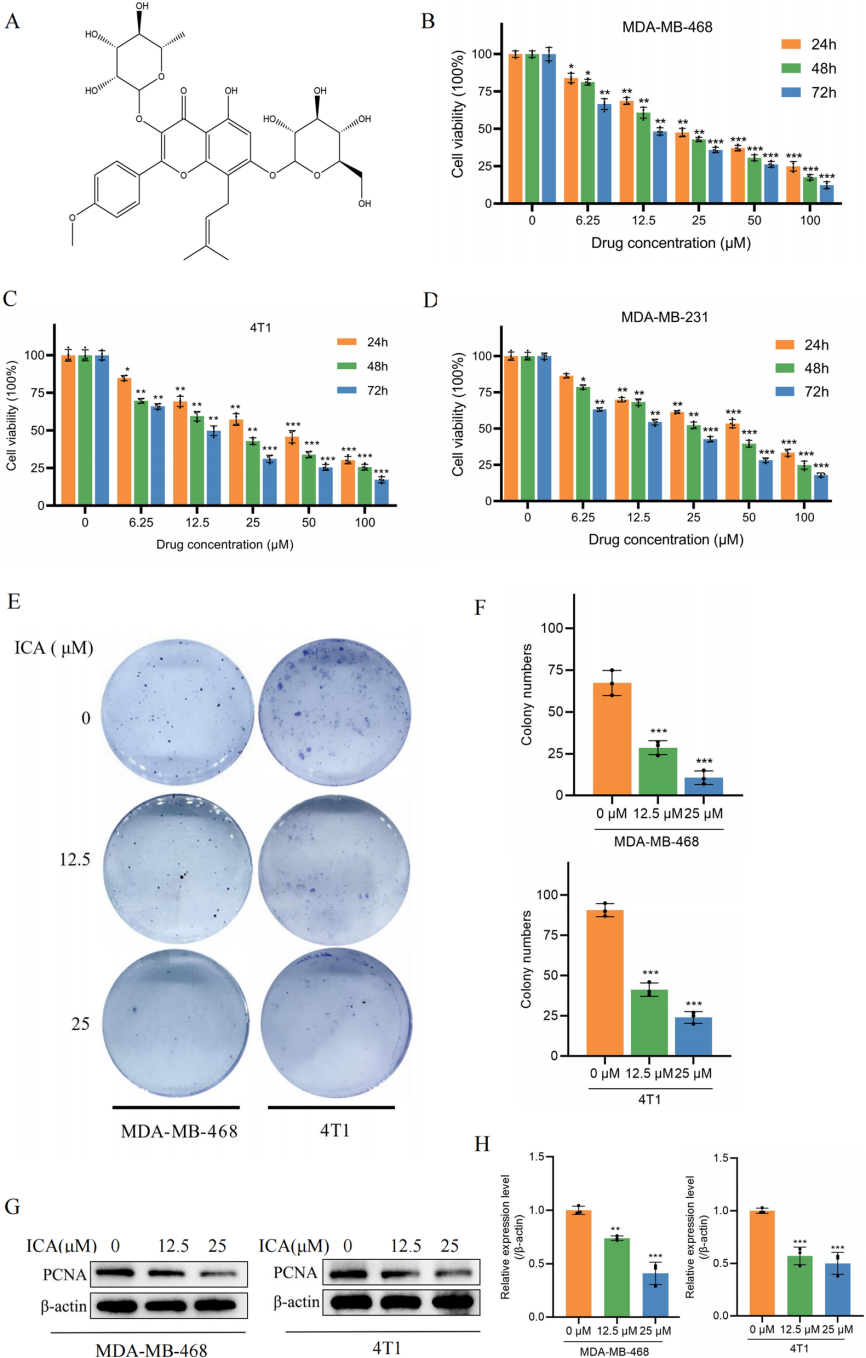
ICA inhibited the growth and proliferation of TNBC cells in vitro. **A** The chemical structure of ICA. **B**–**D** Cell viability analysis using CCK-8 assays of MDA-MB-468, 4T1, and MDA-MB-231 cells following treatment with different concentrations (0, 6.25, 12.5, 25, 50, and 100 µM) of ICA for 24, 48, or 72 h. **E**, **F** Colony formation analysis of 468 and 4T1 cells following treatment with different concentrations (0, 12.5, and 25 µM) of ICA. **G**, **H** The protein expression of PCNA was detected using western blotting with β-actin as an internal control. Bars represent the means ± SD of at least three independent experiments; *P < 0.05, **P < 0.01, and ***P < 0.001 compared with the control group (0 µM). *ICA* icariin, *TNBC* triple-negative breast cancer, *CCK-8* Cell Counting Kit-8, *PCNA* proliferating cell nuclear antigen

The chemical structure of ICA is shown in Fig. 1A. To evaluate the inhibitory effect of ICA on TNBC cells viability, TNBC cell lines 468, 4T1, and 231 were treated with different concentrations of ICA (0, 6.25, 12.5, 25, 50, and 100 µM) for 24, 48, and 72 h. As shown in Fig. 1B–D, ICA significantly reduced the viability of all cells, with 468 (half maximal inhibitory concentration of 27.71, 20.93, and 12.85 µM for 24, 48, and 72 h, respectively) and 4T1 (37.70, 19.74, and 12.45 µM) cells being more sensitive than 231 (47.93, 28.79, and 14.84 µM). The 468 and 4T1 represent human and murine TNBC cell lines, respectively, selected for subsequent studies. The anti-proliferative activity of ICA was verified by colony formation assays and western blot analyses. The results showed that, compared to the control group (0 µM), treatment with ICA led to a significant reduction in colony size and number (Fig. 1E, F), as well as a significantly decreased expression of the PCNA protein (Fig. 1G, H).

### ICA inhibited the migration and invasion of TNBC cells in vitro

**Fig. 2:**
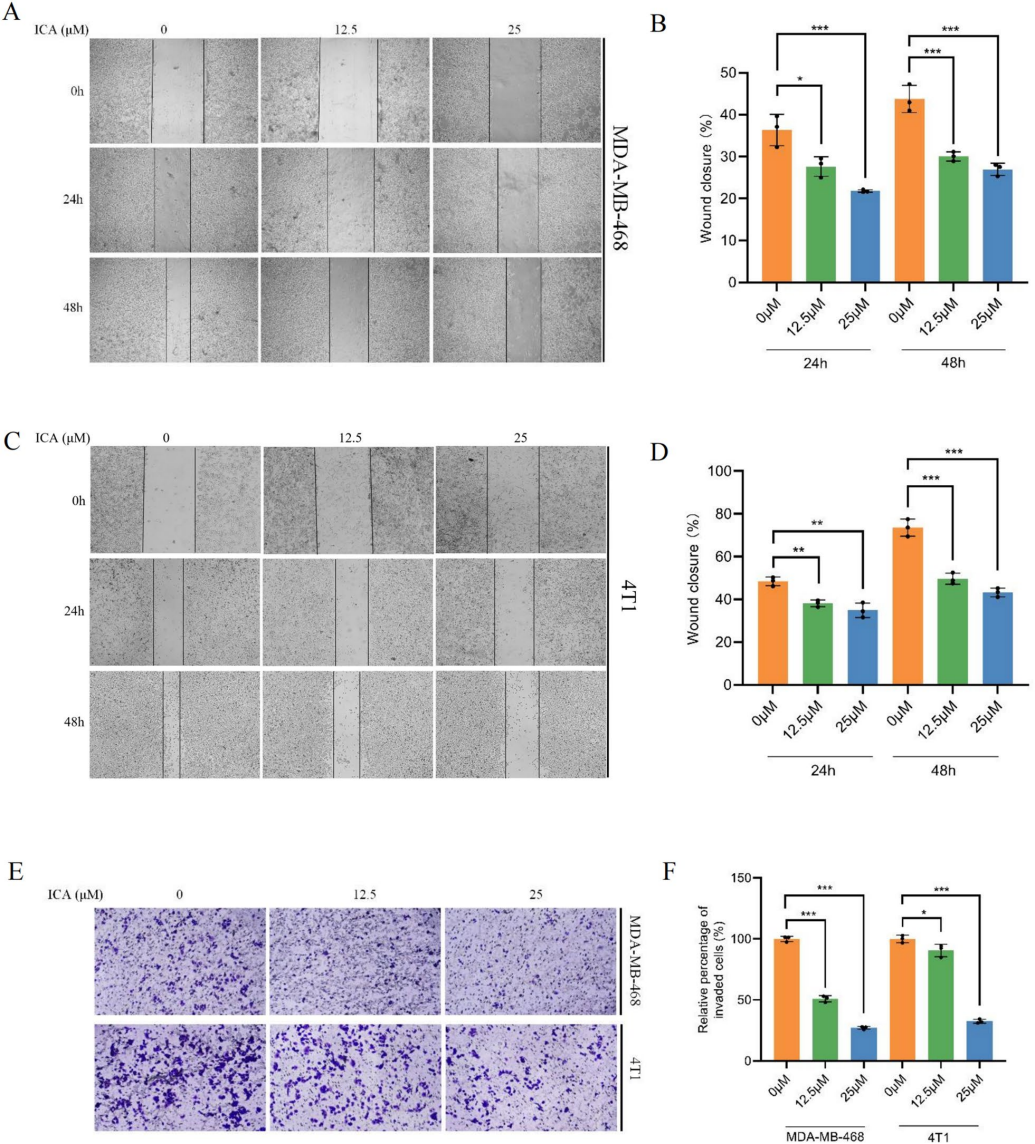
ICA suppressed migration and invasion in TNBC cells. **A**–**D** After treatment with different concentrations (0, 12.5, and 25 µM) of ICA, wound healing of MDA-MB-468 and 4T1 cells was measured. **B**, **D** Analysis of the results of wound healing experiments on MDA-MB-468 and 4T1 cells. **E**, **F** After treatment with different concentrations of ICA, transwell invasion for MDA-MB-468 and 4T1 cells was measured. Bars represent the means ± SD of at least three independent experiments; *P < 0.05, **P < 0.01, and ***P < 0.001 compared with the control group (0 µM). *ICA* icariin, *TNBC* triple-negative breast cancer

Wound-healing assays were used to evaluate ICA's effect on TNBC cell migration. The results showed that the migration of both 468 (Fig. 2A, B) and 4T1 (Fig. 2C, D) cells was significantly inhibited compared with the control group at 24 and 48 h following ICA treatment. Additionally, based on transwell invasion assay results, ICA had a significant anti-invasive effect on both 468 and 4T1 cells in a concentration-dependent manner compared with the control group (Fig. 2D, E).

### ICA induced cell cycle arrest, apoptosis, and autophagy

**Fig. 3:**
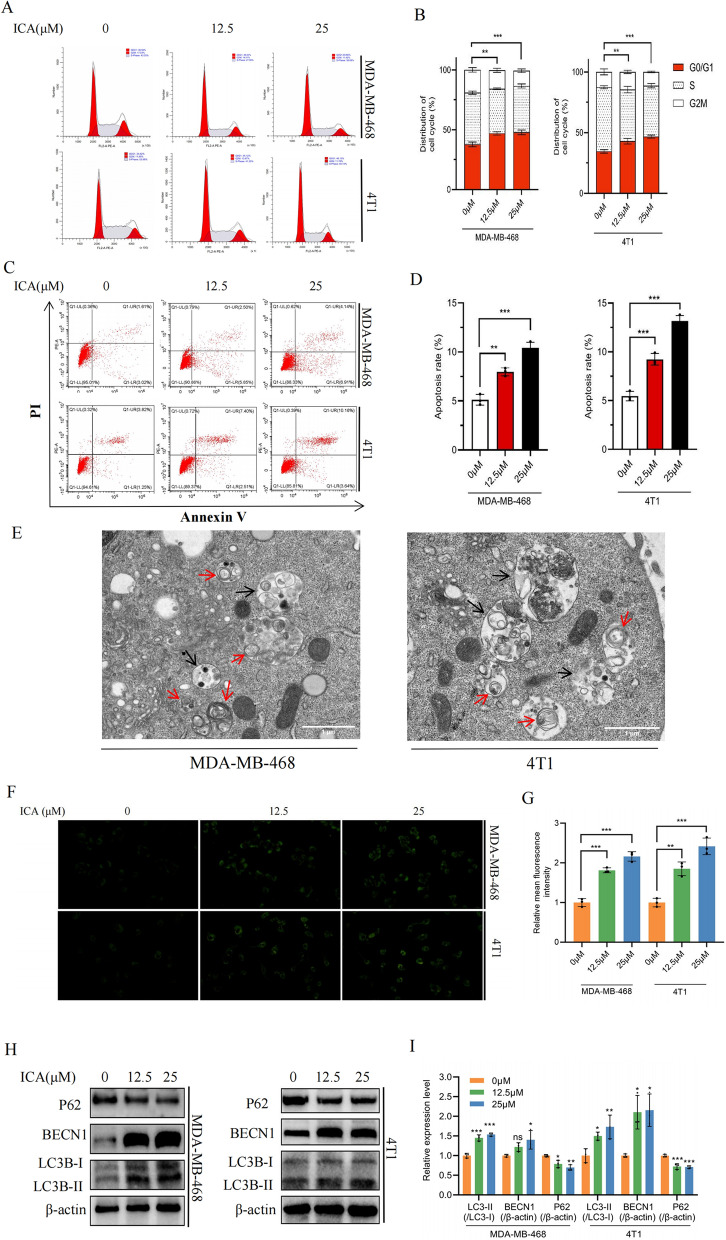
ICA induced cell cycle arrest, apoptosis, and autophagy in TNBC cells. **A**, **B** MDA-MB-468 and 4T1 cells were treated with different concentrations of ICA (0, 12.5, and 25 µM). The cell cycle distribution was analyzed using flow cytometry. **C**, **D** MDA-MB-468 and 4T1 cells were treated with ICA (0, 12.5, and 25 µM) followed by Annexin V/PI double staining and analyzed using flow cytometry. **E** MDA-MB-468 and 4T1 cells were treated with ICA (25 µM) and detected by TEM. The red arrows represent autophagosomes, and the black arrows denote autolysosomes. **F**, **G** MDA-MB-468 and 4T1 cells treated with different concentrations of ICA (0, 12.5, and 25 µM) were stained with MDC and imaged using a microscope (20 ×). The green circles represent autophagosomes. **H**, **I** The protein expression of P62, BECN1, and LC3B was determined using western blotting with β-actin as an internal control. Bars represent the means ± SD of at least three independent experiments; *P < 0.05, **P < 0.01, and ***P < 0.001 compared with the control group (0 µM). *ICA* icariin, *TNBC* triple-negative breast cancer, *TEM* transmission electron microscope, *P62* SQSTM1, *BECN1* Beclin1, *LC3B* MAP1LC3B

As shown in Fig. 3A, B, flow cytometry analysis suggested that the percentage of S-phase cells decreased, while that of G0/G1-phase cells increased following treatment with ICA for 24 h. These results indicated that ICA effectively induced cell cycle arrest in the G0/G1 phase. Additionally, flow cytometric analyses revealed that ICA treatment of TNBC cells for 24 h significantly induced apoptosis in a concentration-dependent manner. To further explore the anti-TNBC activity of ICA, we investigated its effect on autophagy. Transmission electron microscopy (TEM) revealed the presence of autophagic vacuoles and autolysosomes in 468 and 4T1 cells after exposure to ICA for 24 h (red arrows indicating autophagosomes, and black arrows indicating autolysosomes, as shown in Fig. 3E). Additionally, MDC can specifically label autophagosomes through ion trapping and bind to membrane lipids; therefore, it is also often used to detect autophagy [15]. As shown in Fig. 3F, the green fluorescent dots represent the autophagosome cells produced following ICA treatment; the mean fluorescence intensity of autophagosomes was significantly increased compared with that in the control group (0 µM) (Fig. 3G). Certainly, there are three key genes associated with autophagy. LC3B-II indicates autophagic vesicles, BECN1 (Beclin1) coordinates autophagy proteins to autophagic vesicles, and P62 (SQSTM1) degrades within autophagic lysosomes after increased autophagic flux [16]. In 468 and 4T1 cells, ICA treatment resulted in the dose-dependent conversion of LC3B-I to LC3B-II and increased BECN1 expression, whereas P62 protein levels were significantly reduced (Fig. 3H, I).

### ICA promoted autophagy by regulating AMPK/mTOR/ULK1 signaling pathway in TNBC cells

**Fig. 4:**
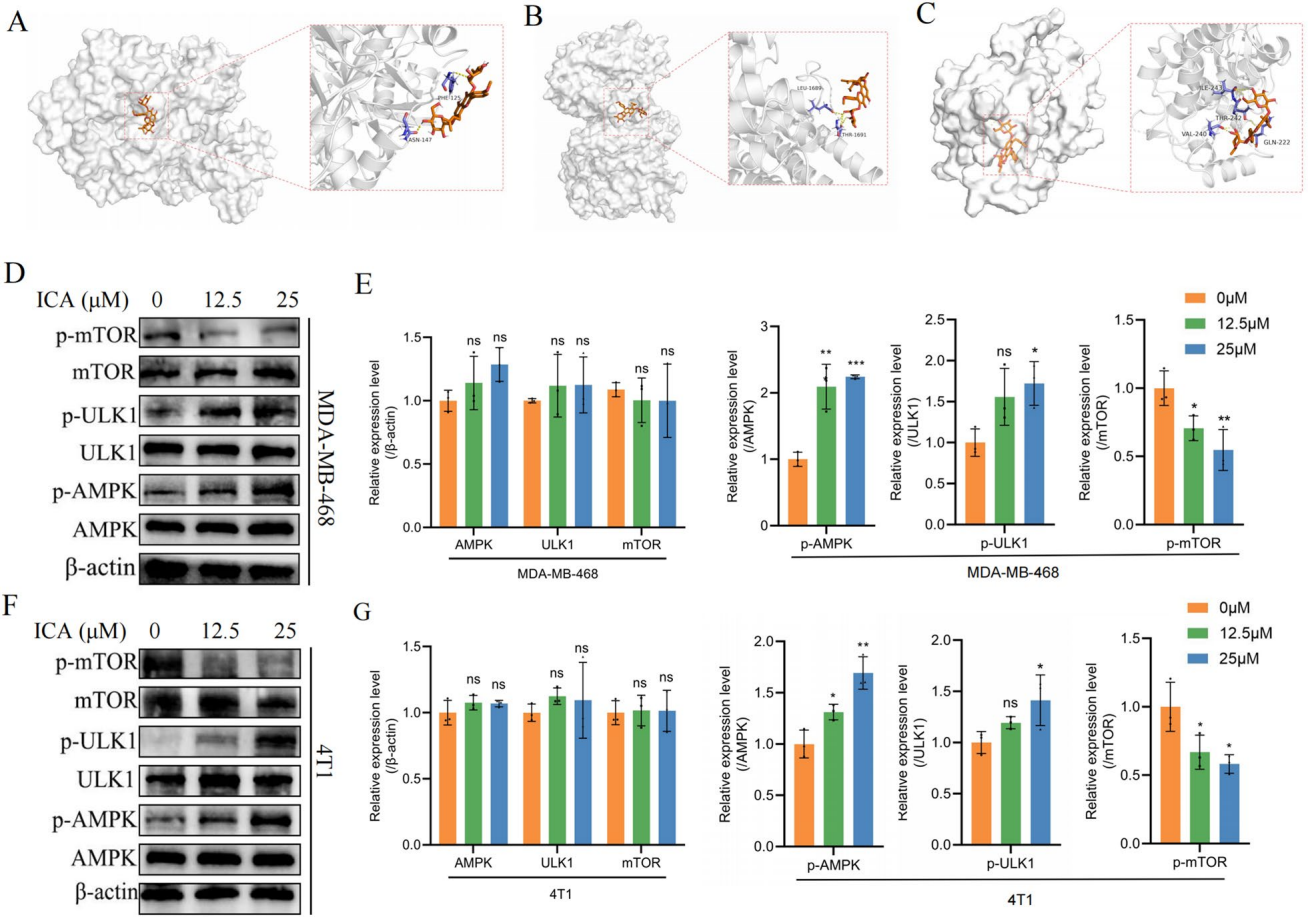
ICA regulated the AMPK/mTOR/ULK1 signaling pathway. **A**–**C** Mode of ICA binding with AMPK, mTOR, and ULK1. The chemical structure in orange indicates ICA, and the purple structure indicates the binding site of ICA, AMPK, mTOR, and ULK1. **D**, **E** The effect of ICA on the expression of AMPK, ULK1, and mTOR and their phosphorylation in MDA-MB-468 cells; **F**, **G** in 4T1 cells. Bars represent the means ± SD of at least three independent experiments; ns, not significant; *P < 0.05, **P < 0.01, and ***P < 0.001 compared with the control group (0 µM). *ICA* icariin, *AMPK* adenosine 5ʹ-monophosphate (AMP)-activated protein kinase, *mTOR* mammalian target of rapamycin, *ULK1* Unc-51-like kinase 1

ULK1 is closely associated with the formation of autophagic vesicles regulated by upstream signaling pathways such as AMPK and mTOR [17]. Therefore, to further validate the role of ICA in the AMPK/mTOR/ULK1 pathway, molecular docking was used to determine whether ICA could act on the three target proteins. Figure 4A–C shows the docking diagrams of ICA with AMPK, mTOR, and ULK1, respectively, with orange representing ICA, purple representing the residues that link the protein to ICA, and yellow representing the hydrogen bonds connecting ICA to the proteins. In Fig. 4D–G, the phosphorylation levels of AMPK and ULK1 increased with escalating doses of ICA treatment, whereas the phosphorylation level of mTOR decreased with increasing doses. These results suggest that ICA could activate the AMPK/mTOR/ULK1 signaling pathway in TNBC cells.

**Fig. 5:**
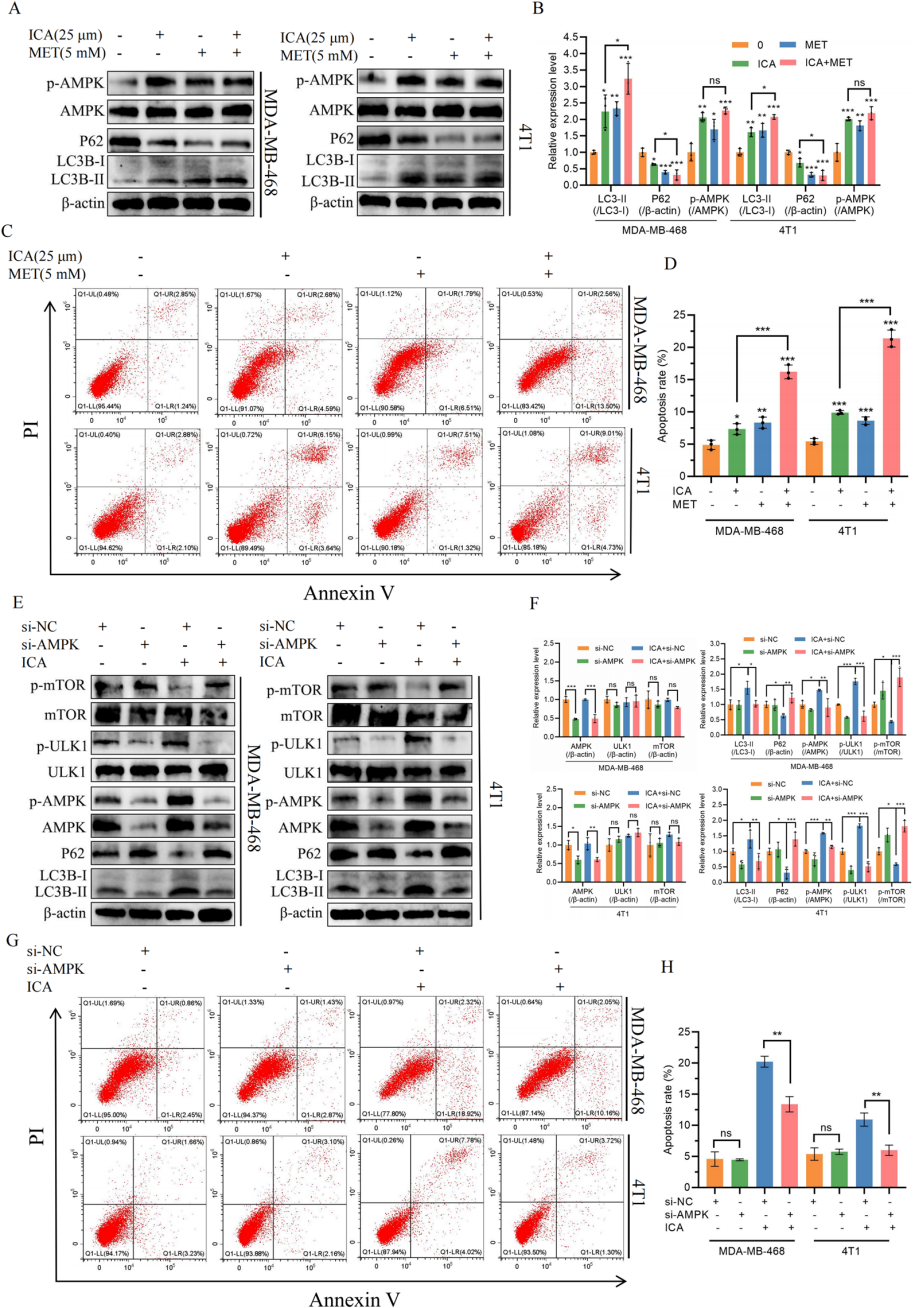
ICA regulated autophagy and apoptosis by acting on AMPK. **A**, **B** Western blotting showing the expression of AMPK, P62, and LC3B proteins in MDA-MB-468 and 4T1 after ICA with or without MET (5 mM) exposure, with β-actin as an internal control. **C**, **D** MDA-MB-468 and 4T1 cells were treated with ICA (25 µM) with or without MET (5 mM). Apoptosis was analyzed using flow cytometry. **E**, **F** The protein expression levels of P62, LC3B, as well as AMPK, mTOR, ULK1, and their phosphorylated proteins were detected using western blotting analyses after knocking down AMPK in TNBC cells. **G**, **H** The apoptosis was analyzed by flow cytometry after knocking down AMPK in TNBC cells. Bars represent the means ± SD of at least three independent experiments; ns, not significant; *P < 0.05, **P < 0.01, and ***P < 0.001. *ICA* icariin, *TNBC* triple-negative breast cancer, *MET* metformin, *P62* SQSTM1, *BECN1* Beclin1, *LC3B* MAP1LC3B, *AMPK* adenosine 5ʹ-monophosphate (AMP)-activated protein kinase, *mTOR* mammalian target of rapamycin, *ULK1* Unc-51-like kinase 1

To further explore the role of AMPK in ICA-induced autophagy and apoptosis, we utilized flow cytometry and western blotting analyses. We compared the therapeutic effects of ICA and MET, a known AMPK and autophagy inducer [18, 19] on TNBC cells. The results indicated that ICA’s impact on AMPK, P62 protein expression, and LC3B-II/LC3B-I ratio (Fig. 5A, B), as well as its modulation of cell apoptosis (Fig. 5C, D), is similar to MET. The combined use of both drugs further enhanced autophagy and apoptosis. Upon siRNA-mediated knockdown of AMPK, ICA treatment in MDA-MB-468 and 4T1 cells resulted in a decrease in the LC3-II/I ratio, an increase in P62 expression, and corresponding changes in mTOR, ULK1, and their phosphorylated proteins (Fig. 5E, F). Additionally, apoptotic cell death significantly decreased (Fig. 5G, H). These data indicated that the AMPK/mTOR/ULK1 pathway plays a crucial regulatory role in ICA-induced autophagy and apoptosis.

### Autophagy inhibitor 3-MA reversed the effect of ICA

**Fig. 6:**
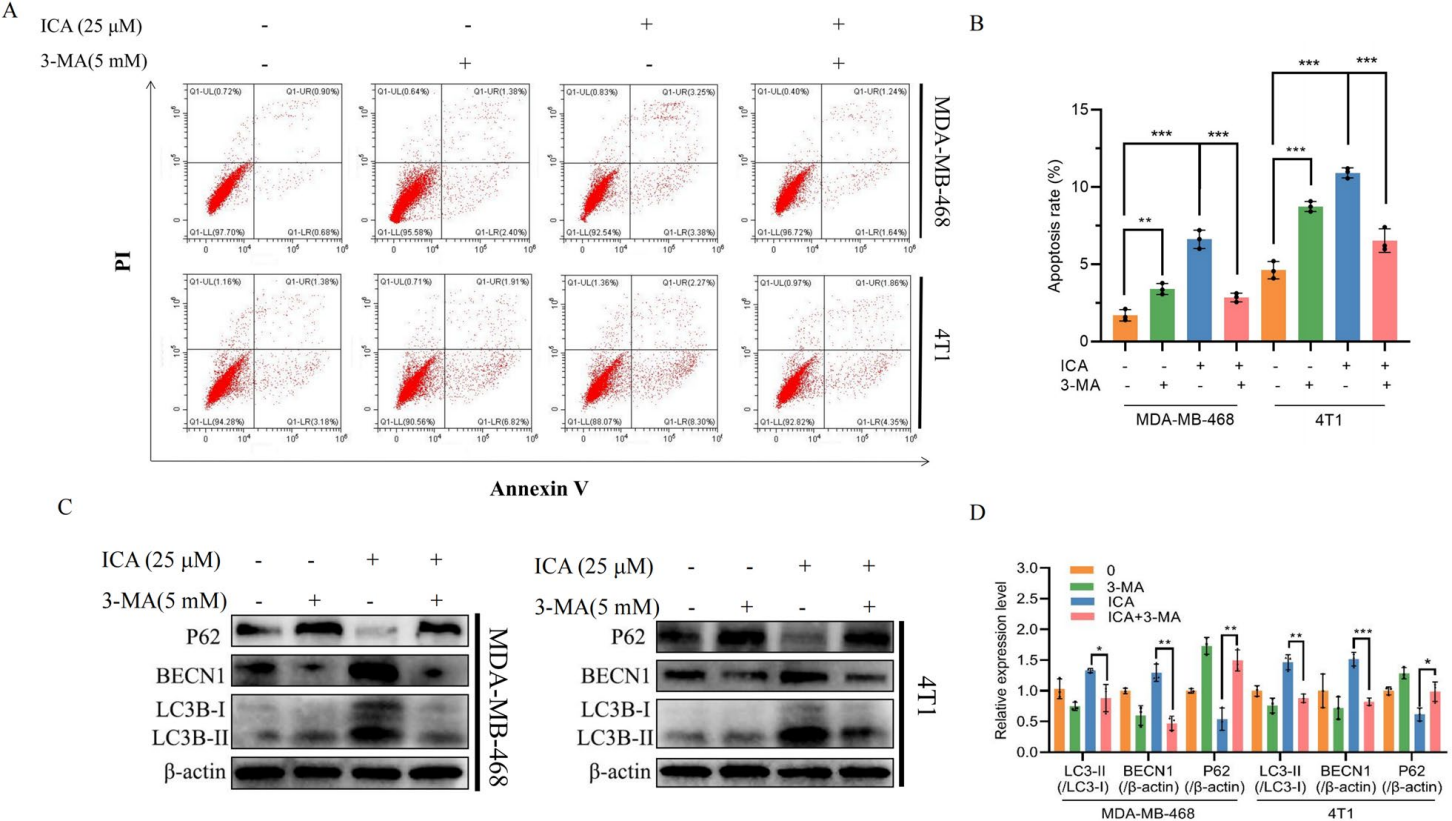
On TNBC cells, 3-MA reversed the effects of ICA. **A**, **B** MDA-MB-468 and 4T1 cells were treated with ICA (25 µM) with or without 3-MA (5 mM). Apoptosis was analyzed using flow cytometry. **C**, **D** Western blotting showing the expression of P62, BECN1, and LC3B proteins in MDA-MB-468 and 4T1 after ICA with or without 3-MA exposure, with β-actin as an internal control. Bars represent the means ± SD of at least three independent experiments; *P < 0.05, **P < 0.01, and ***P < 0.001 compared with the control group (0 µM). *TNBC* triple-negative breast cancer, *3-MA* 3-methyladenine, *ICA* icariin, *P62* SQSTM1, *BECN1* Beclin1, *LC3B* MAP1LC3B

To further investigate the effect of ICA on autophagy in TNBC cells, the autophagy inhibitor, 3-MA, was used in combination with ICA. The addition of 3-MA reduced the proportion of apoptotic TNBC cells (Fig. 6A, B) and reversed the ICA-induced changes in BECN1, LC3B-II/LC3B-I, and p62 protein levels (Fig. 6C, D). These results suggest that ICA exerts its anti-tumor effects by inducing autophagy.

### ICA inhibited tumor growth and promoted autophagy in vivo

**Fig. 7:**
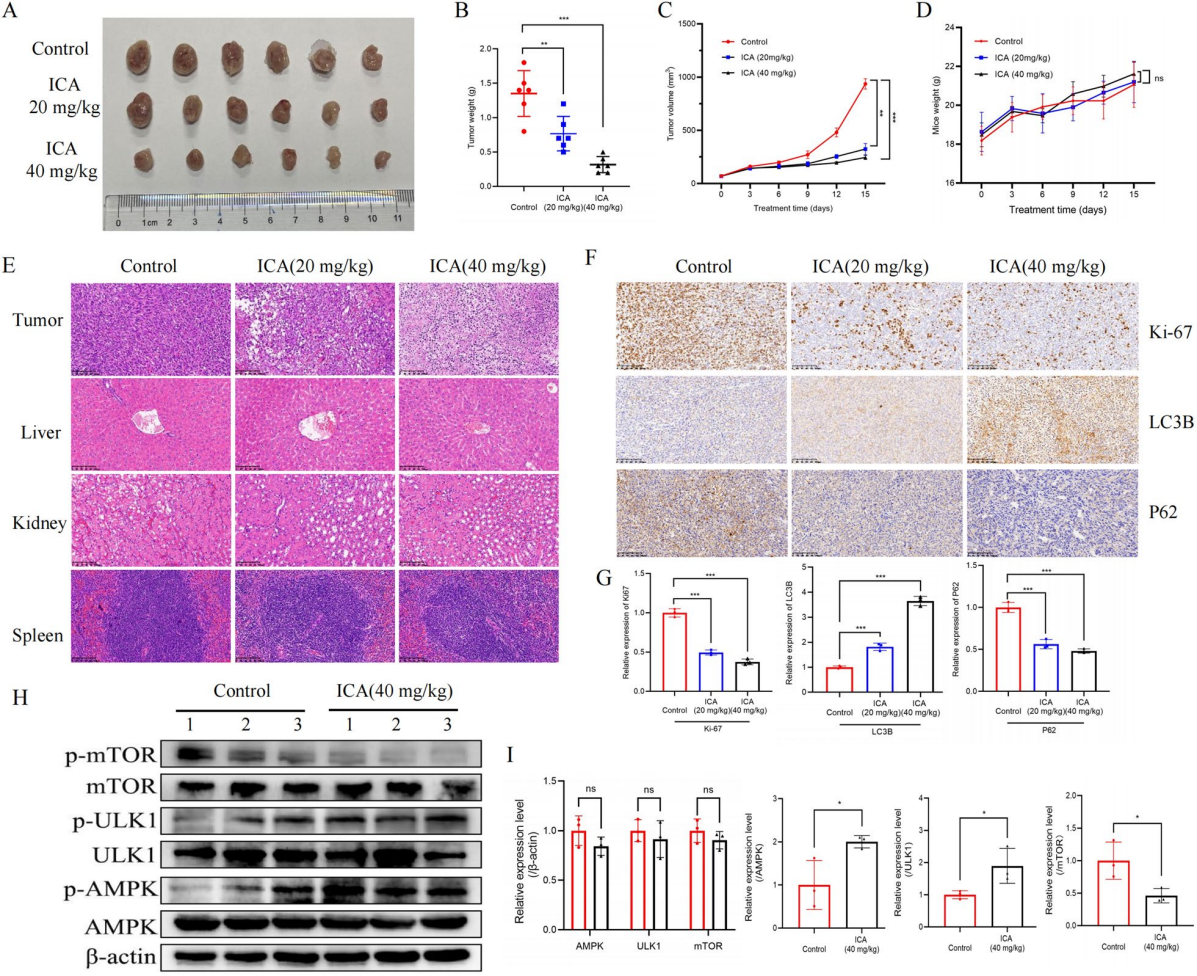
ICA inhibited tumor growth and promoted autophagy in vivo. **A** Image of 4T1 tumors from different groups. **B** Average tumor weight of each group. Bars represent the means ± SD of six mice. **C** The growth of 4T1 xenograft tumors during treatment with control (vehicle), ICA (20 mg/kg), and ICA (40 mg/kg). Bars represent the means ± SD of six mice. **D** Changes in body weight of mice in different groups during treatment. Bars represent the means ± SD of six mice. **E** H&E staining of tumor, liver, kidney, and spleen tissues in each group. **F**, **G** Ki-67, P62 and LC3B immunohistochemical results of tumors from each group. Bars represent the means ± SD of three independent experiments. **H**, **I** The protein expression of p-mTOR, mTOR, p-ULK1, ULK1, p-AMPK, and AMPK in xenograft tumor. Bars represent the means ± SD of three mice tumor samples. *P < 0.05, **P < 0.01, and ***P < 0.001 compared with the control group (0 µM). *ICA* icariin, *TNBC* triple-negative breast cancer, *H&E* hematoxylin and eosin

To determine the antitumor effects of ICA in vivo, 4T1 tumor-bearing mice were treated with 20 or 40 mg/kg ICA. Figure 7A–C shows that ICA treatment significantly inhibited the growth and weight of 4T1 tumors in a dose-dependent manner compared with the control group. The mice’s body weight did not change significantly during treatment compared with the control group (Fig. 7D). H&E staining showed that the tumors in the ICA-treated group showed loosening of the tumor cell layer and coagulation or fragmentation of the nucleus, whereas no significant necrosis was observed in the liver, kidney, or spleen tissues (Fig. 7E). ICA showed no significant side effects and did not cause major organ damage during treatment. The immunohistochemical analyses of the tumors revealed that ICA inhibited nuclear Ki-67-positive cell proliferation, enhanced LC3B expression, and reduced the expression of P62, thereby promoting autophagy and inhibiting proliferation (Fig. 7F, G). The western blotting results of tumor tissue proteins suggested that ICA also increased the phosphorylation of AMPK and ULK1 while reducing the expression of phosphorylated mTOR protein in vivo.

## Discussion

ICA is the main active ingredient of the traditional Chinese medicine Epimedium, which has several therapeutic properties, such as anti-rheumatic [20]. ICA has been shown to have anti-tumor activities in vitro and in vivo. Our results showed that ICA exhibits anti-TNBC properties both in vivo and in vitro. We also found that the anti-cancer effect of ICA was associated with the promotion of autophagy, possibly via ICA activation of the AMPK/mTOR/ULK1 pathway. Our study revealed a novel mechanism of ICA in TNBC associated with tumor autophagy, suggesting ICA is a promising treatment strategy against TNBC.

ICA inhibited the viability and proliferation of MDA-MB-468 and 4T1 cells in a concentration- and time-dependent manner and induced apoptosis. Moreover, the results of the mouse xenograft model experiments showed that ICA significantly inhibited tumor growth while reducing the proliferation index and promoting autophagy. Further studies on the mechanism of ICA killing of TNBC cells revealed that autophagic vesicle production was increased in ICA-treated TNBC cells. Additionally, with higher ICA doses, the LC3-BII/LC3B-I ratio increased (an indicator of autophagic flux), BECN1 expression increased (a marker of autophagosome formation), and p62 expression decreased (an indicator of autophagic degradation) [21]. Autophagy has emerged as a potential target for cancer therapy [22, 23]. Several small molecules target autophagy and may, therefore, be used for TNBC treatment. For example, SLLN-15 (an oral selenopurine molecule), LYN-1604 (a ULK1 agonist), and flubendazole exert anti-tumor effects by inducing autophagy [24–26].

Autophagy and apoptosis, two programmed cell death pathways, have been focal points in cancer research. These two metabolic pathways play crucial roles in maintaining cellular and organismal homeostasis [27]. Ideally, autophagy and apoptosis contribute to tumor suppression, as autophagy aids in eliminating cancer cells, whereas apoptosis prevents their survival [28, 29]. However, as cancer progresses, autophagy exhibits a dual role due to its crosstalk with the regulatory mechanisms of apoptosis. For one thing, autophagy itself serves as a form of cell death known as autophagic cell death. Specific inhibition of autophagy by suppressing, depleting, or deleting various crucial autophagy-related genes and/or proteins could prevent cell death, which proves that the ultimate cell death process is caused by autophagy rather than apoptosis [30, 31]. For another, the induction of autophagy promotes the activation of apoptosis. Autophagic proteins play an additional role in the transduction of pro-apoptotic signals, finally leading to cell death by inducing apoptosis [32, 33]. Additionally, there is another situation where autophagy inhibits apoptosis. This is generally achieved by clearing damaged mitochondria, increasing the threshold for apoptosis induction, and selectively reducing the abundance of pro-apoptotic proteins in the cytoplasm [32, 34]. In simple terms, autophagy is a metabolic pathway in most eukaryotic cells that promotes both cancer cell survival (protective autophagy) and death (cytotoxic/non-protective autophagy) in different types and stages of cancer [35]. Therefore, whether autophagy is beneficial or inhibitory to therapeutic outcomes is context-dependent. To further investigate the impact of ICA on autophagy and apoptosis, we conducted experiments using the autophagosome formation inhibitor 3-MA. The results showed treatment with 3-MA inhibited the effect of ICA, suggesting that autophagy regulates TNBC cell apoptosis.

An increasing body of evidence suggests a paradoxical role of autophagy in response to anticancer treatments, much like its capacity to either trigger cell death or enhance cell survival. One perspective is that autophagy is activated as a protective mechanism, mediating the acquisition of acquired resistance in certain cancer cells during chemotherapy. Another perspective is that autophagy may also function as an executioner, with anticancer treatment enhancing autophagic cell death [36]. In some preclinical models, autophagy inhibition synergistically enhances cytotoxicity with several anticancer drugs. For instance, the combination of chloroquine with 5-fluorouracil increases its anti-colorectal cancer effect, and co-administration with topotecan enhances its anti-lung cancer effect [37, 38]. In contrast, some studies have found that inducing autophagic cell death is an alternative method to kill tumor cells without developing resistance to anticancer drugs. The synergistic promotion of autophagy is observed when autophagy inducers are combined with cytotoxic drugs. For example, the combination of cannabinoids (autophagy inducers) and temozolomide (TMZ) strongly activates autophagy-mediated cancer cell death, resulting in a potent antitumor effect against both TMZ-sensitive and TMZ-resistant tumors [39]. Therefore, the role of autophagy in cancer treatment is not straightforward; it may vary depending on cell types, stress signals, and other circumstances. Areas that need to be further addressed through experiments include elucidating the impact of the tumor microenvironment on autophagic function and determining the role of autophagy in regulating treatment sensitivity.

**Fig. 8:**
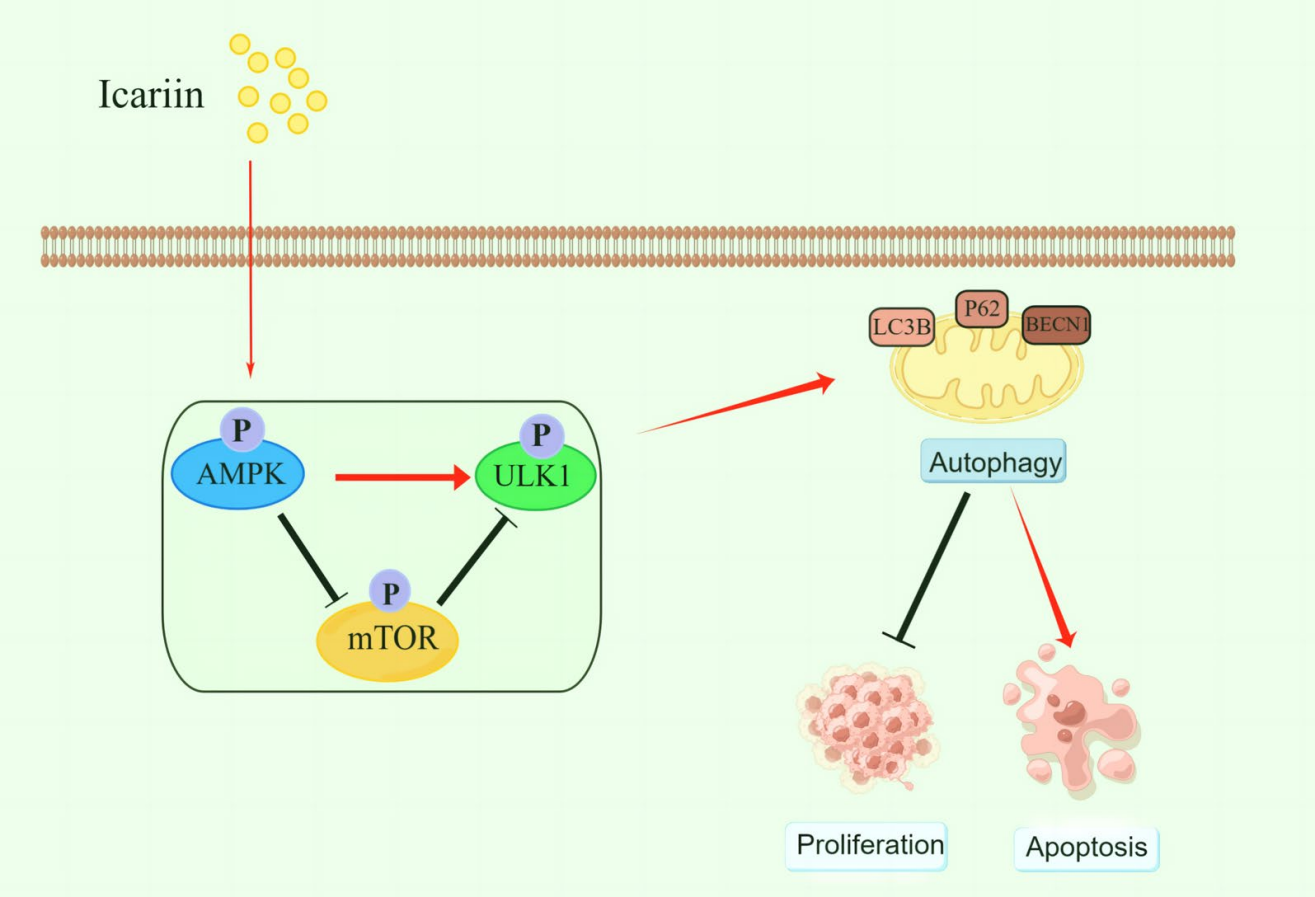
Schematic illustration of the ICA effects on TNBC. *ICA* icariin, *TNBC* triple-negative breast cancer

The AMPK/mTOR/ULK1 signaling pathway plays an important role in regulating autophagy. In this study, we used molecular docking technology to predict whether ICA could act on these three targets. Western blotting results suggested that ICA treatment increased the phosphorylation of AMPK and ULK1 in TNBC cells and decreased the phosphorylation of mTOR. Subsequently, we utilized si-RNA to knock down AMPK in cells, reversing ICA’s effects on cell autophagy and apoptosis. As a key regulator of energy metabolism, cell growth, and autophagy, AMPK can be activated in various ways, including reactive oxygen species (ROS) [40, 41]. A previous study showed that ICA increases ROS production and activates mitochondrial apoptosis pathways [14]. mTOR, which regulates cell growth and metabolism, negatively regulates autophagy activation [42]. ULK1, a serine/threonine kinase, induces autophagosome formation [43]. In this canonical pathway, AMPK directly phosphorylates and activates ULK1; mTOR is a key regulator of autophagy as it inhibits ULK1 activation [44]. The results of the molecular docking experiments showed that ICA can act on various targets, and ULK1 can be activated both directly by phosphorylated AMPK and indirectly by mTOR inactivation, thereby affecting autophagy (Fig. 8). Understanding the interplay between ICA, the AMPK/mTOR/ULK1 signaling pathway, and autophagy can provide insights into the mechanisms underlying the therapeutic effects of ICA in TNBC. Targeting this pathway and modulating autophagy may provide new opportunities for the development of combination therapies to improve the outcomes of patients with TNBC.

This study has some limitations. Firstly, the study did not investigate the pharmacokinetics and pharmacodynamics of ICA, and therefore, its safety and efficacy as a potential therapeutic agent remain to be investigated. Secondly, the influence of ICA on the sensitivity of TNBC to chemotherapy drugs has not been investigated and is currently under investigation for further refinement. Lastly, meticulously planned randomized controlled trials have the potential to yield crucial clinical insights and establish a more robust theoretical framework for the clinical implementation of ICA in TNBC management. Consequently, additional research endeavors are necessary to delve deeper into the role of ICA in TNBC treatment and unravel its underlying mechanism.

## Conclusions

Our study demonstrated the potent anti-TNBC efficacy of ICA both in vivo and in vitro and revealed the molecular mechanism by which ICA induces autophagy to inhibit TNBC progression by activating the AMPK/mTOR/ULK1 signaling pathway. These findings suggest the potential of ICA as a therapeutic agent for the treatment of TNBC. Further studies are warranted to verify our findings, identify molecular targets, and overcome the limitations of experimental models. This study provides novel insights into the mechanism of action of ICA and will help to facilitate future research and development of TNBC-targeted therapies.

## Data Availability

Data will be made available on request.

## Abbreviations

BC: Breast cancer
TNBC: Triple-negative breast cancer
ICA: Icariin
FDA: Food and Drug Administration
TNBCs: Triple-negative breast cancers
H&E: Hematoxylin and eosin
IHC: Immunohistochemistry
DAB: Diaminobenzidine tetrahydrochloride
SD: Standard deviation
AMPK: Adenosine monophosphate-activated protein kinase
ULK1: Unc-51-like kinase 1
mTOR: Mammalian target of rapamycin
